# 4565 Sex-Specific Differences in the Genomic Landscape of Pediatric and Adult Glioblastoma

**DOI:** 10.1017/cts.2020.343

**Published:** 2020-07-29

**Authors:** Nikolay A. Ivanov, Nadia Dahmane, Jeffrey P. Greenfield, Christopher E. Mason

**Affiliations:** 1 Clinical and Translational Science Center, Weill Cornell; 2 Weill Cornell Medicine

## Abstract

OBJECTIVES/GOALS: It has been previously shown that pediatric high-grade glioma (pHGG) survival is different between sexes. We set out to find out whether there are sex-specific differences in the genomic landscapes of pHGG that may underlie this sex disparity. METHODS/STUDY POPULATION: We downloaded Illumina 450k DNAm data from ArrayExpress and GeneExpressionOmnibus. The *minfi* package was used to process raw DNAm data. Sex chromosomes and CpGs that are common SNPs were removed. Surrogate variables (SVs) were estimated via the *sva* Bioconductor package. Differentially methylated CpGs were identified by fitting a multiple linear regression model for the DNAm level at each CpG, with independent variables being sex (a binary variable) and the estimated SVs. RNAseq data was downloaded from Cavatica, and differential gene expression analysis was carried out via the *DESeq2* package. RESULTS/ANTICIPATED RESULTS: In the pediatric glioblastoma (GBM) DNAm data [58 female & 91 male IDH wt samples; ages 0.1–21 yrs;], we found 7,371 differentially methylated cytosines (DMCs) at FDR≤0.05. Of the DMCs, 289 had DNAm differences between male and female samples ≥10%. The majority of probes (68%) were in CpG islands, shelves, or shores. We also found 4 differentially methylated regions (DMRs) between sexes (FWER≤0.1). In the adult GBM DNAm samples [32 F & 32 M IDH wt samples; ages 22–75 yrs], we found only 117 DMCs at FDR≤0.05, and no DMRs. In the RNAseq dataset [68 F & 54 M pHGG samples, ages 0.08–30.6 yrs], we found 383 differentially expressed genes (at FDR≤0.05), and 16 of them (4%) overlapped a DMC. DISCUSSION/SIGNIFICANCE OF IMPACT: Our findings demonstrate that pHGG exhibits sex-specific methylome differences. Interestingly, this difference is greater in the pediatric population as compared to adults. The pHGG transcriptome also differs by sex, which may be related to differential DNAm in a minority of cases.

